# Sea Urchin Skin Lesions: A Case Report

**DOI:** 10.5826/dpc.1102a09

**Published:** 2021-03-08

**Authors:** María Florencia Suarez-Conde, María Gabriela Vallone, Virginia Mariana González, Margarita Larralde

**Affiliations:** 1 Dermatology Department, Hospital Alemán, Buenos Aires, Argentina

**Keywords:** aquatic dermatoses, echinoderm, sea urchin, dermoscopy, dermatoscopy, foreign body

## Introduction

Sea urchins are invertebrates and members of the echinoderm family that are found on the rocky seabeds and corals in tropical and temperate waters. There are over 700 known species of sea urchins, 80 of which contain toxic substances to humans. As a result, they are responsible for a wide range of conditions, including simple penetrating wounds, granulomatous processes, and systemic complications [1].

We present the case of a patient who was affected by a penetrating injury caused by sea urchin spines. The diagnosis was established based on the epidemiological characteristics, clinical presentation, and dermoscopic features.

## Case Presentation

A 20-year-old man presented with puncture wounds on his right sole after having stepped on a rock in the sea on the coast of Italy. The patient was referred with localized pain during the first 48 hours.

**Figure 1:**
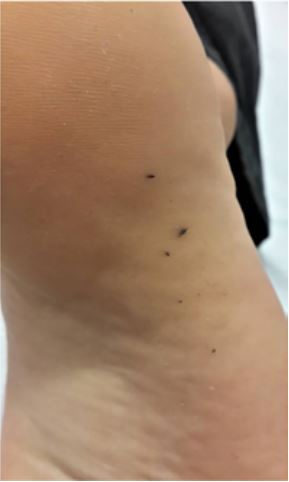
Cylindrical foreign bodies in the sole and ankle of the right foot.

**Figure 2:**
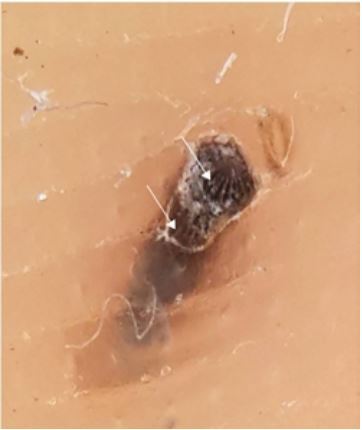
Lateral view of one of the spines with a violaceous-black pigmentation and a striated external surface that correlates with the white lines seen on dermoscopy.

**Figure 3:**
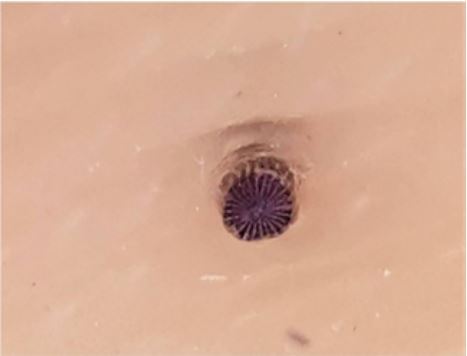
Frontal view of one of the fragments, with circular shape and regular whitish and violaceous lines, radiating towards the periphery.

Physical examination revealed several cylindrical foreign bodies exhibiting blackish pigmentation in the right sole and ankle (Figure 1). Polarized dermoscopy revealed foreign bodies, some of them placed in an oblique angle within the stratum corneum and showing a conical shape, a violaceous-black pigmentation, and a striated external surface. Others, placed transversely to the skin, exhibited a circular shape with concentric whitish lines, regularly radiating towards the periphery and matching the external striae or septa (Figures 2 and 3). These dermoscopic images bear a remarkable resemblance, although with less magnification and resolution, to scanning electron microscopy images of sea urchin spines [2].

Clinical and dermoscopic findings and epidemiological features established the diagnosis. The spines were then mechanically removed.

## Conclusions

Skin disorders caused by sea urchin contact most commonly compromise feet and ankles. The spines can penetrate the skin and easily break into several fragments, and the most common initial manifestation is acute localized pain that resolves within hours or days, as was the case of our patient [1]. Diagnosis is usually based on clinical findings and history. Differential diagnoses include other types of foreign bodies such as wood fragments and puncture wounds from sharp objects, among others. Histopathological examination may also prove helpful in cases of late granulomatous reactions [1]. According to our findings, dermoscopy may also serve as a valuable tool. Indeed, dermoscopic images of sea urchin spines were remarkably similar to electron microscopy images published in the literature.
